# Retinal Vessel Segmentation: An Efficient Graph Cut Approach with Retinex and Local Phase

**DOI:** 10.1371/journal.pone.0122332

**Published:** 2015-04-01

**Authors:** Yitian Zhao, Yonghuai Liu, Xiangqian Wu, Simon P. Harding, Yalin Zheng

**Affiliations:** 1 Department of Eye and Vision Science, University of Liverpool, Liverpool, United Kingdom; 2 Department of Computer Science, Aberystwyth University, Aberystwyth, United Kingdom; 3 School of Computer Science and Technology, Harbin Institute of Technology, Harbin, China; 4 St Paul’s Eye Unit, Royal Liverpool University Hospital, Liverpool, United Kingdom; University of Iowa, UNITED STATES

## Abstract

Our application concerns the automated detection of vessels in retinal images to improve understanding of the disease mechanism, diagnosis and treatment of retinal and a number of systemic diseases. We propose a new framework for segmenting retinal vasculatures with much improved accuracy and efficiency. The proposed framework consists of three technical components: Retinex-based image inhomogeneity correction, local phase-based vessel enhancement and graph cut-based active contour segmentation. These procedures are applied in the following order. Underpinned by the Retinex theory, the inhomogeneity correction step aims to address challenges presented by the image intensity inhomogeneities, and the relatively low contrast of thin vessels compared to the background. The local phase enhancement technique is employed to enhance vessels for its superiority in preserving the vessel edges. The graph cut-based active contour method is used for its efficiency and effectiveness in segmenting the vessels from the enhanced images using the local phase filter. We have demonstrated its performance by applying it to four public retinal image datasets (3 datasets of color fundus photography and 1 of fluorescein angiography). Statistical analysis demonstrates that each component of the framework can provide the level of performance expected. The proposed framework is compared with widely used unsupervised and supervised methods, showing that the overall framework outperforms its competitors. For example, the achieved sensitivity (0:744), specificity (0:978) and accuracy (0:953) for the DRIVE dataset are very close to those of the manual annotations obtained by the second observer.

## Introduction

The human retina is a light sensitive tissue lining the inner surface of the eye. This tissue is extremely rich in blood vessels for its high physiological demands and dysfunction of the retinal vasculature can result from several diseases [1]. Vascular abnormalities can be seen in various retinal diseases. Study of the retinal circulation is of great importance in the management of retinal diseases, but also provides unique opportunity to study the microvascular damage to the brain in cerebral malaria [2]. Structural changes in the retinal vasculature may also indicate hypertension, stroke, heart disease and nephropathy [3]. The retina is visible to examination and accessible to high-resolution, non-invasive imaging. This provided a unique window that allows direct visualization and analysis of the inner retinal vascular circulation for studying various related conditions. Automated analysis of the retinal vasculature becomes an active research area in the field of medical imaging for its diagnostic and prognostic significance.

Our application concerns the automated detection of retinal blood vessels in diagnostic retinal images such as color fundus images and fluorescein angiography images. The automated detection of blood vessels is a prerequisite in the development of automated system for the analysis of vessels. Recent years have witnessed the rapid development of methods for retinal vessel segmentation, as evidenced by extensive reviews [4, 5]. For the purpose of this paper this list is intended only to provide readers with some insight into this problem domain, and is by no means exhaustive. Most existing methods are automated techniques without interaction from the user during the segmentation. However, we noted that interactive segmentation techniques, such as Live Vessel [6], were proposed for improving the segmentation performance. Broadly speaking, all the established automated segmentation techniques may be categorized as either supervised segmentation [7–13] or unsupervised segmentation [14–23] with respect to the overall system design and architecture.

Supervised segmentation requires hand-labeled gold standard images for training, and each pixel is represented by a feature vector which is obtained from local or global information of the image. The prerequisite for this approach is that a set of features having the necessary discriminative ability have to be extracted for training and classification processes. These features can be extracted by different filters: for example, the Gabor filter used in [8]. Various classifiers can be used for the classification tasks including k-nearest neighbors [7], support vector machine (SVM) [9, 13], artificial neural networks (ANN) [24], Gaussian mixture models (GMM) [11], or AdaBoost [10], to name only a few.

In contrast, unsupervised segmentation refers to methods that achieve the segmentation of blood vessels without using training data, or explicitly using any classification techniques. This category includes most segmentation techniques in the literature, such as [16–18], and our framework as described in this paper. The unsupervised segmentation techniques may be further divided into two classes: kernel-based and tracking-based methods.

Various kernels (or filters) have been designed to enhance the vessels in an image for the ease of segmentation. Most of them are based on image intensity, such as matched filter [25, 26], steerable filters, amplitude-modified second order Gaussian filter [27], eigenvalue-based filter [28], multi-scale linear operators [20], wavelet [12, 17], Gabor filters [8], COSFIRE filters [22, 29] and so on. These intensity-based filters are susceptible to intensity inhomogeneity and will encounter further problems when they are required faithfully to enhance vessels of different scales. On the other hand, a filter based on local phase information of an image is emerging and seems to be able to avoid the problems met by the intensity based filters [18].

In the tracking-based methods, vessels are seen as lines, and these methods try to follow vessel edges by exploiting their local information. Various vessel profile models, such as Gaussian profile [30, 31], generic parametric model [32], Bayesian probabilistic model [33], and mutliscale profile [34], have been used to find the path which has the best matches to the vessel profile model.

There has been increasing interest in using active contour models for the purpose of vessel segmentation [16, 18, 35]. The twin-ribbon model [16] is a parametric active contour model that has the disadvantages of being difficult to formulate and slow. The curve evolution model adopted by [18] is slow in convergence and requires good initialization to start with. Region-based active contour models, such as the well-known Chan-Vese (CV) model [36], have recently become popular [35]. The region-based models do not require edge information to guide the segmentation, instead making use of the region information. In their original work, Chan and Vese proposed using the level set implementation to solve the energy minimization problem. The level set method is slow due to its iterative nature, and requires re-initialization during the iterations.

In general there are three major challenges to be addressed in automated retinal vessel segmentation:

- First, image quality is often an issue of concern for the development of automated segmentation. Existing segmentation techniques still face challenges in segmenting the entire vessel structures accurately and automatically, due to poor contrast, inhomogeneous backgrounds and presence of noise during image acquisition.
- Second, the complexity of vascular structure (e.g., multiple scales and orientations), the high degree of anatomical variation across the population and the complexity of the surrounding tissue/organs, pose significant challenges in vessel segmentation. Enhancement of vessels is an effective way to facilitate segmentation: but commonly used enhancement filters are sub-optimal in terms of performance.
- Third, an efficient and robust segmentation model is desirable. It has become very difficult to choose an optimal model, or to identify a single set of optimal parameters for a particular segmentation method that will work across a variety of data.

Being well acquainted with the above three challenges, we have developed a new framework that seamlessly integrates three distinct technical components, with the underlying idea that each of these techniques will address one of the above challenges. More specifically, these state-of-the-art components, namely Retinex, local phase based enhancement and graph cut-based active contour model, are used in sequence to build an efficient, accurate and robust segmentation framework.

The rest of this paper is organized as follows: Section Methods describe the proposed segmentation framework in detail. In particular, an image-wise enhancement method based on Retinex theory is presented for illumination correction. The enhancement of vessels by means of using local phase information is then introduced, followed by descriptions on the graph cut-based active contour model that is used to achieve the segmentation from the enhanced maps produced by local phase enhancement. A brief introduction to the four datasets that are used for the purpose of evaluation, and to the evaluation metrics used are provided in Section Datasets and Evaluation Metrics. Section Results present the experiments and results. Finally, the paper is concluded in Section Discussion and Conclusions.

## Methods

The proposed segmentation framework comprises three major steps (each with a distinct component): Retinex-based inhomogeneity correction, local phase-based enhancement and graph cut-based active contour segmentation. These steps will be described in turn below.

### Retinex-based Inhomogeneity Correction

Intensity inhomogeneity, often inherited from the retinal image acquisition process, poses a significant challenge to many image processing tasks. To this end, image enhancement or inhomogeneity correction for the captured images is necessary, with a view to removing any effects of varying illumination conditions.

Being a well-known global enhancement method, histogram equalization considers the frequencies of colors and intensities of pixels in an image and then re-assigns these properties. It can be easily implemented and is effective for images with colors and intensities concentrated in a narrow band. However, it cannot handle those images with colors and intensities spanning the whole range of display devices. Another widely used global enhancement method, gamma correction, has some success in enhancing images that are either too dark of too bright, however, the best choice of the parameter gamma is dependent on the image under consideration. This explains why the contrast-limited adaptive histogram equalization (CLAHE) algorithm [37] is often used to improve the local contrast to avoid the inhomogeneous regions in retinal image analysis [22, 38, 39]. However, this method enhances the image uniformly irrespective of whether a region is in the foreground or background.

On the other hand, Land and McCann [40] proposed an interesting idea named Retinex theory, whereas the *Retinex* is a combination of the words *retina* and *cortex*. The Retinex theory shows that the color constancy involves not just human perception, but also human visual processing and interpretation. Adapted from the field of computer vision, Retinex is used to remove unfavorable illumination effects from images in order to improve their quality. For instance, it has been used to remove unwanted illumination effects from color or gray images to improve their quality [41], and to enhance the retinal image for artery/vein classification [42].

In the Retinex theory, a given image *I* can be modeled as a component-wise multiplication of two components, the reflectance *R* and the illumination *L*: *I* = *R* * *L*. Typically, the reflectance image reveals the object of interest more objectively, as such it can be regarded as the enhanced version of image *I*. A number of local based methods have been proposed for estimating the *R* and *L*. Usually, the smoothing approaches are adopted to estimate the decomposition components. Jobson et al. [41] used the transformation of the ratio of the original image and Gaussian smoothed intensity of a pixel to determine the reflectance *R*. Park et al. [43] introduced an iterative adaptive smoothing method to estimate the illumination *L*, and the weight of each pixel is obtained by a coefficient combining the functions of gradients and inhomogeneities of the pixel.

In this work, we proposed to use the Retinex theory based on bilateral filter implementation for image inhomogeneity correction. The reasons for employing a bilateral filter are two-fold. It is an edge-preserving smoothing filter that can maintain the edge information essential for accurate vessel detection [43, 44]. Moreover, the bilateral filter has been confirmed to be effective in recent work on image decomposition [44].

Let *x* be a pixel of an image *I*. The reflectance image *R*(*x*) can be obtained by taking the difference between the logarithms of the original image *I*(*x*) and the resulting image *L*(*x*) after applying a bilateral filter to the original *I*(*x*). This is given as:

$$\begin{array}{c} R(x)=log(I(x)+1)-log(L(x)+1). \end{array}$$ (1)

*L*(*x*) can be written as:

$$\begin{array}{c} L(x)=M^{-1}\left(x\right)\int_{W}I(\ell )g(\ell ,x)s(\ell ,x)d\ell , \end{array}$$ (2)

with the normalization factor *M* given as

$$\begin{array}{c} M\left(x\right)=\int_{W}g(\ell ,x)s(\ell ,x)d\ell , \end{array}$$ (3)

where *g*(ℓ, *x*) measures the spatial closeness between a pixel *x* and a nearby pixel ℓ in window *W* (a window size of 3 × 3 is used in this paper), and function *s*(ℓ, *x*) measures the similarity of the intensities between *x* and ℓ. Both the geometric measurement *g* and similarity function *s* are Gaussian functions of the Euclidean distance between their arguments. More specifically, *g* and *s* are defined as follows, respectively:

$$\begin{array}{c} g(\ell ,x)=e^{-\frac{1}{2}{\left(\frac{d(\ell ,x)}{\sigma_{d}}\right)}^{2}}, \end{array}$$ (4)

$$\begin{array}{c} s(\ell ,x)=e^{-\frac{1}{2}{\left(\frac{d\left(I(\ell ),I(x)\right)}{\sigma_{r}}\right)}^{2}}, \end{array}$$ (5)

where *σ*_*d*_ shows the spatial spread based on the desired amount of low-pass filtering, and *σ*_*r*_ is the geometric spread of the image intensity range, which is set to achieve the desired amount of combination of intensity values. The *σ*_*r*_ and *σ*_*d*_ are empirically chosen (both are 0.3 in this paper).

**Fig 1:**
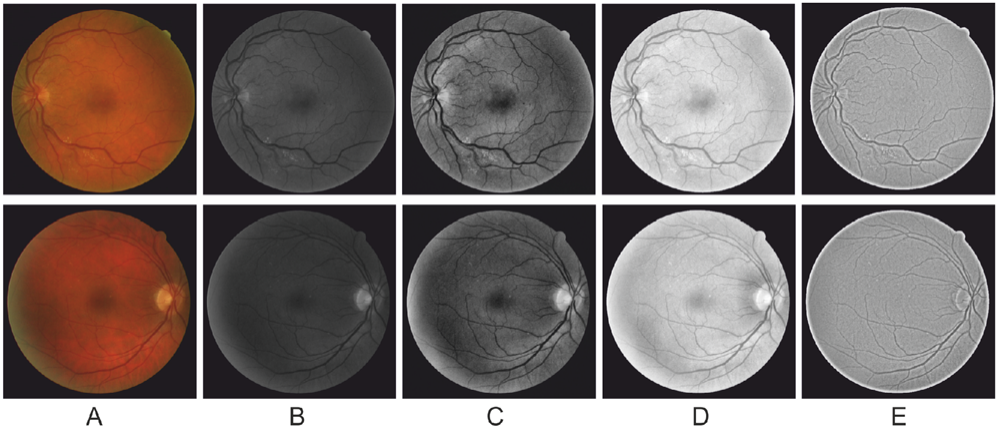
A comparative study on image-wise enhancement techniques. (A) Two example images from the DRIVE dataset. (B) The green channel of (A). (C) and (D) show the results of applying Histogram Equalization and Gamma correction image enhancement methods on (B), respectively. Each method enhanced image contrast: however, there still exist large areas of inhomogeneity. (E) Results after applying Retinex on (B). Retinex enhances the contrast between vessels and background well, and in consequence the vessels are more easily identifiable.

In essence, the bilateral filtering replaces the intensity value at *x* with an average of similar and nearby intensity values. In the smooth regions, intensity values within a small neighborhood are similar to each other, and the filtered intensity will not differ significantly. Therefore, the bilateral filtering averages away small, weakly correlated differences of intensity. The normalization term *M* ensures that the weights add up to one for all nearby neighboring intensity values. As a result, the filter replaces the large intensity value at the center of a given neighborhood by an average of the large intensity value in its vicinity, and vice versa. Following the Retinex theory, we normalized *R*(*x*) to the range of [0, 1]. Fig. 1 shows some example results produced by our Retinex approach and by other enhancement techniques. Overall, it seems that all the methods successfully enhance the contrast of the vessels, whilst the Retinex also corrects the inhomogeneities within the image (the optic disk and foveal area are corrected as well).

### Local Phase-based Vessel Enhancement

Local phase, together with local energy and local orientation, is an important local feature of an image. It can be viewed as a measure of structural information (e.g. lines and edges) of an image. As such, local phase plays increasingly important roles in a wide range of applications, such as edge detection, symmetry analysis, and registration tasks. More recently, it has been shown that this information can be used to enhance linear (or tubular) structures in a more precise way, and to produce promising results in vessel segmentation problems [18]. However, its performance has not been thoroughly evaluated on large datasets against more established enhancement filters, such as [17]. In this work, this will be one of the tasks we seek to perform. It is worth noting that local phase and local energy are often used interchangeably, following convention, here this filter is still referred to as a ‘local phase-based’ filter only, even though it has been modulated by the local energy.

For a one-dimensional (1*D*) problem, local phase can be estimated by the Hilbert transform under the concept of analytical signal. For problems with two-dimensions (2*D*) or higher, it may be estimated by using quadrature filters under the concept of monogenic signals.

A quadrature filter comprises a pair of even and odd filters with phase difference of *π*/2. Let $E_{n}^{j}$ and $O_{n}^{j}$ denote the even symmetric and odd-symmetric parts of a quadrature filter at scale *n* and orientation *j*. At each point *x* of an image *I*, the filter response $q_{n}^{j}(x)$ is given by $q_{n}^{j}=e_{n}^{j}(x)+o_{n}^{j}(x)i$, $i=\sqrt{-1}$, while $e_{n}^{j}(x)=I(x)*E_{n}^{j}$ and $o_{n}^{j}(x)=I(x)*O_{n}^{j}$ respectively, where * denotes a convolution operation. Multiple orientations are needed to capture structures (e.g. vessels) present in different directions.

The local energy $A_{n}^{j}(x)$ and local phase $\varphi_{n}^{j}(x)$ at scale *n* and orientation *j* are defined respectively as follows:

$$\begin{array}{c} A_{n}^{j}\left(x\right)=\sqrt{e_{n}^{j}{\left(x\right)}^{2}+o_{n}^{j}{\left(x\right)}^{2}}, \end{array}$$ (6)

and

$$\begin{array}{c} \varphi_{n}^{j}\left(x\right)=\arctan\left(\frac{o_{n}^{j}\left(x\right)}{e_{n}^{j}\left(x\right)}\right). \end{array}$$ (7)

It is clear that at edges, $o_{n}^{j}(x)$ has the maximal response while $e_{n}^{j}(x)$ is almost 0, while at lines $o_{n}^{j}(x)$ is almost 0 and $e_{n}^{j}(x)$ has the maximal response. This suggests that image edges align with the zero crossing of the real part of the phase map. In order to avoid confusion caused by changes on structural direction, for the imaginary part we will use the absolute value of the imaginary part $o_{n}^{j}$, so that ${\bar{q}}_{n}^{j}=e_{n}^{j}+|o_{n}^{j}|i$.

Filters at each scale for all directions have to be combined to obtain a rationally invariant phase map. The response at scale *n* is defined as $q_{n}=\sum_{j=1}^{J}{\bar{q}}_{n}^{j}$, where *J* is the number of directions under consideration. In this paper, for each scale four filters of directions (0, *π*/4, *π*/2, and 3*π*/4) are used.

**Fig 2:**
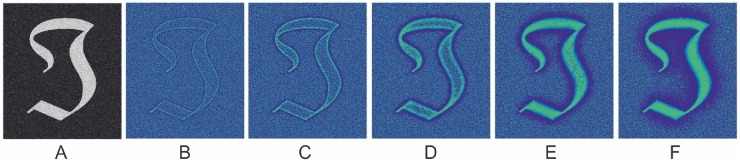
Illustration of the enhancement effect on a test image using the local phase filter at different scales from 1 to 4.

In order to enhance all the structures in a given image, multiple scales will be needed (2 or 3 are suggested by [18], and have been demonstrated in Fig. 2). The filter response at each scale is weighted by *βth* power of the magnitude of the filter response vector at that scale. The sum of these weighted differences is then normalized by the sum of the magnitude of the filter response vectors over all scales. This produces the following equation:

$$\begin{array}{c} P=\frac{\sum_{n=1}^{N}q_{n}{\left|q_{n}\right|}^{\beta }}{\sum_{n=1}^{N}{\left|q_{n}\right|}^{\beta }}, \end{array}$$ (8)

where *N* is the number of scales. *β* is the order number of the power of the magnitude of the filter response vector at each scale. There are many quadrature filters that might be used [45], but here we will stay with the optimized log-norm filter for its optimal performance in both spatial and frequency domain [46]. More specifically, the center frequency is 5*π*/7, the bandwidth is 2 octaves, and the filter has a size of 15 × 15.

The zero-crossing of the real part indicates the edges. Consequently, only the real part is used in the enhancement of the vessels. Following Lathen’s work [18], *P* is normalized in order to make the map more regular for segmentation purposes and to minimize noise. The final ‘vesselness map’, ℒ𝒫, is defined as follows:

$$\begin{array}{c} ℒ𝒫=\text{real}\left\{\frac{P\cdot |P|}{{\left|P\right|}^{2}+a^{2}}\right\} \end{array}$$ (9)

This vesselness map has some unique properties. It has a positive value inside the lines (or vessels) but a negative value in the background. As designed, it has a zero value at the edge of the line structures. This vesselness map needs further processing to segment the vessels.

Fig. 2(A) illustrates an image of a symbol mimicking vessels with varying width and orientation where Gaussian noise (*σ* = 0.2) is added to the image. The filtering results of using 4 scales with *β* = 1 are shown in Fig. 2(B)-(E) respectively. Fig. 2(F) demonstrates the final result, obtained by combining all the four discrete filtering results. It can be observed that the final result illustrates a clear line structure with distinct edges.

In practice, it was noted that some filters, such as eigenvalue-based filter and COSFIRE [29], may produce lower response at the end of vessels than in the middle of a vessel. Azzopardi et al. attempted to address this problem by introducing asymmetric B-COSFIRE filter [22]. From Fig. 2(B)-(F), however, it can be seen that without any special treatment the local phase based filter achieves strong responses at the end of the vessel-like symbol, and performed equally to the other vessel-like regions.

**Fig 3:**
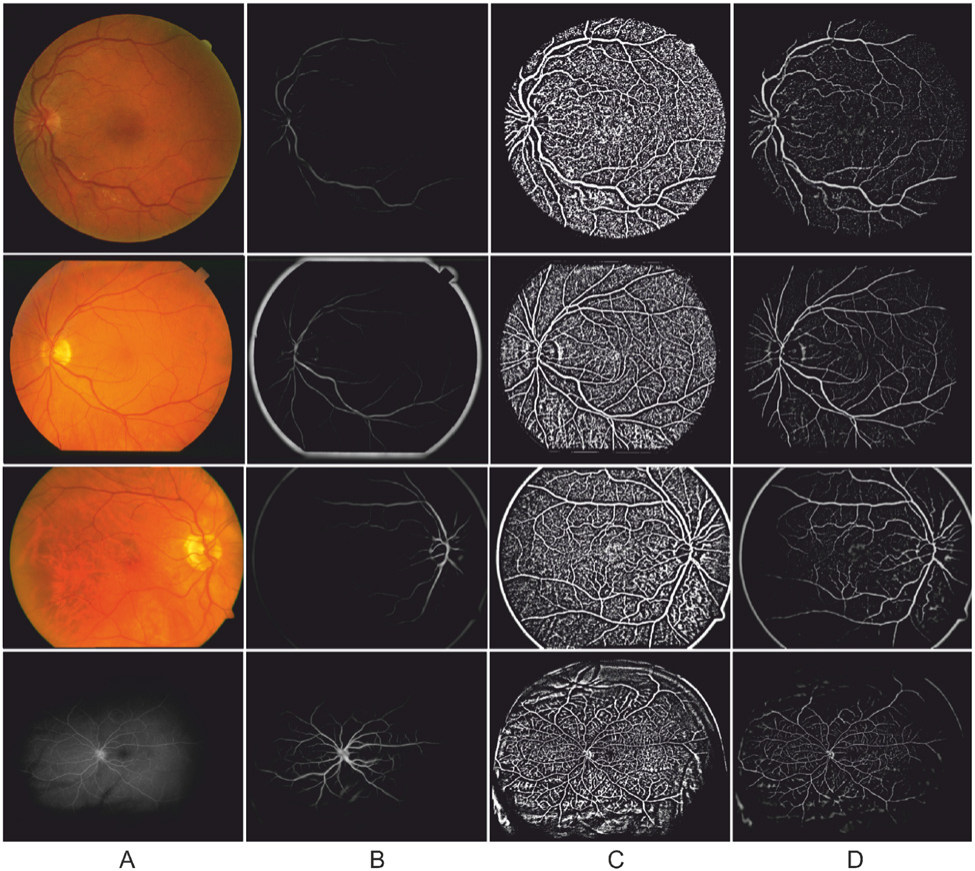
Enhancement results produced by an eigenvalue-based method [28], a wavelet-based method [17] and the local phase method, respectively. An image was randomly chosen from each of the four datasets. From top to bottom: DRIVE, STARE, ARIA, and VAMPIRE. (A) Example images. (B) Eigenvalue-based enhancement results. (C) Wavelet-based enhancement results. (D) Local phase based enhancement results.

In this paper, two other common enhancement methods were chosen for comparative study: Eigenvalue-based [28] and Wavelet-based [17]. For reproducibility, the parameters used on these filters were: *Eigenvalue-based scales: 1−8, scale ratio: 2. Wavelet scales used: 2−3*. Note, these free parameters may be adjusted to produce better results according to the nature of images. However, the above mentioned parameters were recommended values in literature [47] and [17], respectively. Fig. 3 demonstrates the results after applying three different enhancement methods: Eigenvalue-based [28], Wavelet-based [17], and the local phase method. One example image as shown in Fig. 3(A) is randomly chosen from each of the DRIVE, STARE, ARIA, and VAMPIRE datasets (for more details about these datasets see Section Datasets). Illustrative enhancement results are shown in Fig. 3(B)-(D).

**Fig 4:**
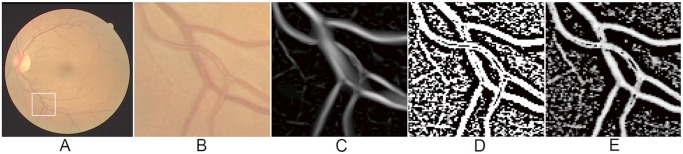
Enhancement results on selected region with vascular bifurcation and crossover produced by an eigenvalue-based method [28], a wavelet-based method [17] and the local phase method, respectively. (A) A randomly chosen image from the DRIVE dataset. (B) Selected region with vascular bifurcation and crossover. (C) Eigenvalue-based enhancement results. (D) Wavelet-based enhancement results. (E) Local phase based enhancement results.

Fig. 4 shows the enhanced result from a selected region containing both vascular bifurcations and crossovers by three different filters. It clearly can be seen that the bifurcation and crossover regions are poorly enhanced by the eigenvalue-based filter (Fig. 4(C)), which made them less distinguishable compared with normal vessel regions. On the other hand, the wavelet-based filter and local phase-based filter, as show in Fig. 4 (D)-(E), can produce consistent results at the bifurcation and crossover region when compared to the other parts of the vessels. Visually the local phase enhanced vesselness map seems more pleasing.

### Graph Cut-based Active Contour Method

Intuitively, given the vesselness map generated from the local phase enhancement, the segmentation may be done if all the edge points (zero-crossing) can be located by a method as simple as a thresholding approach. Unfortunately this is not the case in real applications: for example, a thresholding approach cannot guarantee smooth boundaries of the structure. In light of this inadequacy, more sophisticated segmentation techniques will be needed for the best results. On the other hand, the computation cost of a segmentation tool is also an important factor to be taken into account for potential real applications. For these two reasons, we advocate here a graph cut-based active contour without edge model [48].

The well-known active contour without edge model, or simply the CV model [36], is one the most influential segmentation models in the literature. The CV model aims to divide an input image into two regions with a smooth boundary and low intra-region intensity variance. Let Ω be a bounded and open set in *R* ^*n*^ (*n* = 2 for a two-dimensional image for example). Without loss of generalizability, a given image *I* can be viewed as a discrete sample of a continuous image function *I* = *I*(*x*,*y*) that has values at any point (*x*,*y*) ∈ Ω. The aim of segmentation is to partition Ω into two regions Ω_*i*_, *i* = 1,2, where $\Omega_{2}=\Omega \setminus \bar{\Omega_{1}}$. Let Γ denote the boundary that separates the two regions.

As proposed by Chan and Vese [36], a segmentation problem can be formulated as an energy minimization problem:

inf_Γ,*c*_1_,*c*_2__ *E*(Γ,*c*_1_, *c*_2_),

where

$$\begin{array}{ccc} E(\Gamma ,c_{1},c_{2}) & = & \mu \cdot \text{Length}(\Gamma )\\ & & +\;\lambda_{1}\int_{inside(\Gamma )}{\left(I-c_{1}\right)}^{2}dxdy+\lambda_{2}\int_{outside(\Gamma )}{\left(I-c_{2}\right)}^{2}dxdy, \end{array}$$ (10)

and *c*_1_(*x*, *y*) and *c*_2_(*x*, *y*) is the mean intensity in Ω_1_ and Ω_2_, respectively. In the original paper *λ*_1_ = *λ*_2_ = *λ*: we keep them distinct here for the purposes of generalization.

By introducing a level set function *ϕ*(*x*,*y*), the above equation can be rewritten as

$$\begin{array}{ccc} E(\phi ,c_{1},c_{2}) & = & \mu \int_{\Omega }\delta \left(\phi \right)\left|\nabla \phi \right|dxdy+\lambda_{1}\int_{\Omega }{\left(I-c_{1}\right)}^{2}H\left(\phi \right)dxdy\\ & & +\;\lambda_{2}\int_{\Omega }{\left(I-c_{2}\right)}^{2}\left(1-H\left(\phi \right)\right)dxdy, \end{array}$$ (11)

where *H*(*x*) and *δ*(*x*) are the Heaviside and Dirac function, respectively. The minimization of the above equation can be obtained by decoupling the variables. The resulting nonlinear partial difference equation was solved with a semi-implicit method [36], which is unconditionally stable and can also be solved numerically using other similar finite differences schemes. For the actual numerical implementation please refer to the original paper [36]. We refer to this method as the LS method in the following sections.

For the level set implementation, re-initialization of *ϕ* is required during the iterations, and the convergence is often slow. With a view to addressing this issue, the CV model has been continuously improved. In particular, a total variation model has become popular. Under the total variation framework, Equation 11 can be re-written as

$$\begin{array}{ccc} E(u,c_{1},c_{2}) & = & \mu \int_{\Omega }\left|\nabla u\right|dxdy+\lambda_{1}\int_{\Omega }u{\left(I-c_{1}\right)}^{2}dxdy\\ & & +\;\lambda_{2}\int_{\Omega }\left(1-u\right){\left(I-c_{2}\right)}^{2}dxdy, \end{array}$$ (12)

where *u* is a membership function, 0 ≤ *u* ≤ 1. The object (or foreground) can be determined by setting *u* > 0.5. This formulation can be solved in an elegant manner by using the dual projection algorithm [49]. We denote this method as the TV method in the following sections.

More recently, in light of its computational efficiency, graph cut approach has been exploited to cope with the above minimization problem. The CV model can be approximated and optimized under the graph cut framework [48]. This model is adopted here to segment the local phase enhanced map and is denoted as the GC method.

Let 𝒩 be the set of edges {(*i*, *j*)}, and ℳ denote the number of image pixels. The discrete energy function of Equation 12 can be given as:

$$\begin{array}{c} E\left(x\right)=\sum_{\{i,j\}\in 𝒩}E_{ij}\left(x_{i},x_{j}\right)+\sum_{i=1}^{ℳ}E_{i}\left(x_{i}\right), \end{array}$$ (13)

where *x* = (*x*_1_, ⋯, *x*_𝒩_) is the binary labelling in which the *x*_*i*_ is either 0 or 1, depending on whether the pixel *i* belongs to the background or foreground.

The first term approximates the regularization term (term 1 in Equation 12) while the second term here approximates the region terms (term 2 and 3 in Equation 12). The unary term *E*_*i*_ and binary term *E*_*ij*_ are defined as:

$$\begin{array}{c} E_{i}^{0}=\lambda_{1}{\left(I_{i}-c_{1}\right)}^{2},E_{i}^{1}=\lambda_{2}{\left(I_{i}-c_{2}\right)}^{2} \end{array}$$ (14)

$$\begin{array}{c} E_{i,j}=\left\{\begin{array}{cc} \mu w_{ij},\; & \mathrm{if}{𝓍}_{i}\neq {𝓍}_{j}\\ 0,\; & \mathrm{otherwise}. \end{array}\right. \end{array}$$ (15)

where $E_{i}^{0},E_{i}^{1}$ denote the weights between the node *i* and the two terminals, *I*_*i*_ is the intensity value at pixels *i*. *w*_*ij*_ denotes the weight between neighboring pixels *i* and *j*. The Euclidean length of the boundary separating Ω_1_ and Ω_2_ is used to define *w*_*ij*_, as suggested by [50]:

$$\begin{array}{c} w_{ij}=\frac{\delta^{2}\cdot \Delta \phi_{ij}}{2\cdot |e_{ij}|}, \end{array}$$ (16)

where *δ* is the cell-size of the grid, |*e*_*ij*_| is the Euclidean length of the edge *e*_*ij*_, and the angle *ϕ*_*ij*_ is restricted to the interval [0,*π*].

## Datasets and Evaluation Criteria

We have employed four public retinal image datasets for the purpose of evaluation of our segmentation framework. These datasets are chosen primarily because of the availability of reference standard from manual annotations of the retinal vessels by experts. All the images in these four datasets are centered at the macula, the center of the retina. In this section, we will first provide a brief introduction to these datasets, followed by an introduction to the evaluation metrics that were used in our experiments.

### Datasets

**DRIVE** (Digital Retinal Images for Vessel Extraction): consists of a total of 40 color fundus photos, obtained in the course of a diabetic retinopathy screening program in the Netherlands. The images were acquired using a Canon CR5 non-mydriatic 3-CCD camera (Canon, Tokyo, Japan) with a 45 degree field of view. Each image resolution is 768×584 pixels. The set of 40 images was divided into a test and a training set, each containing 20 images. The DRIVE dataset is available at http://www.isi.uu.nl/Research/Datasets/DRIVE/.

**STARE** (STructured Analysis of the Retina): conceived and initiated at the University of California. This database contains 20 color photographic images of the fundus, 10 of which show evidence of pathology. The digitized slides were captured by a Topcon TRV-50 fundus camera (Topcon, Tokyo, Japan), and the photos were digitized to 605×700 pixels. The STARE dataset is available at http://www.ces.clemson.edu/~ahoover/stare/.

**ARIA** (Automated Retinal Image Analysis). The dataset consists of three groups: the first group has 92 images showing age-related macular degeneration (AMD), the second group has 59 images from patients with diabetes, and the third consists of 61 images of healthy eyes. The images were collected by the St Paul’s Eye Unit and the University of Liverpool. All fundus images were taken using a Zeiss FF450+ fundus camera (Carl Zeiss Meditec, Inc., Dublin, CA). The images were captured at a resolution of 768×576 pixels. The ARIA dataset is available at http://www.eyecharity.com/aria_online.html.

**VAMPIRE**: this dataset comprises eight ultra-wide field of view images acquired with the OPTOS P200C camera (Optos PLC, Dunfermline, UK). Four of the images are from a sequence of an AMD retina, while the other four are from a healthy retina. Each image captures about 200 degrees of the retina and has a size of 3,900×3,072 pixels [51].

### Evaluation Metrics

Four commonly-used metrics were employed to evaluate the performance of the competing methods: sensitivity, specificity, accuracy, and the area under a receiver operating characteristic (ROC) curve, also known as *AUC*. Sensitivity is a measure of effectiveness in identifying pixels with positive values: specificity performs the same function for pixels with negative values. Accuracy indicates the overall segmentation performance. These metrics are defined as follows:

$$\begin{array}{c} \mathrm{sensitivity}(\mathrm{Se})=\frac{tp}{tp+fn}, \end{array}$$ (17)

$$\begin{array}{c} \mathrm{specificity}(\mathrm{Sp})=\frac{tn}{tn+fp}, \end{array}$$ (18)

$$\begin{array}{c} \mathrm{accuracy}(\mathrm{Acc})=\frac{tp+tn}{tp+fp+tn+fn}, \end{array}$$ (19)

where *tp*, *tn*, *fp* and *fn* indicate the true positive (correctly identified vessel pixels), true negative (correctly identified background pixels), false positive (incorrectly identified vessel pixels), and false negative (incorrectly identified background pixels), respectively.

In essence, vessel segmentation can be viewed as an imbalanced data classification problem, in which there are typically much fewer vessel pixels than the background pixels. In such a case accuracy (*Acc*) will be skewed by the dominant classes, while *AUC* on the other hand has the ability to reflect the trade-offs between the sensitivity and specificity. As suggested by [52], the *AUC* can be derived as

$$\begin{array}{c} \mathrm{AUC}=\frac{Se+Sp}{2}, \end{array}$$ (20)

Note that an *AUC* of 0.50 means that the classification is equivalent to a pure random guess, and an *AUC* of 1.0 means that the classifier distinguishes class examples perfectly.

Statistical analysis was performed in order to evaluate the effect of different factors, including choice of dataset, vessel enhancement filters and segmentation programs on the *AUC* and computational time. For the purposes of this analysis we have grouped all the experiment results together. Analysis of variance (ANOVA) with Tukey post hoc analysis was performed using the SPSS version 21.0 (SPSS Inc., Chicago, IL, USA). A *p* value of 0.05 is considered statistically significant.

## Results

In this section we performed experiments to evaluate the performance of our proposed framework. We first evaluated the effect of individual components, such as the Retinex enhancement, vessel enhancement and segmentation methods, on the performance of the proposed framework across all four datasets, and then compared our method with several popular methods in the literature on the DRIVE and STARE datasets only. For the DRIVE dataset, the manual segmentations from set A are used as ground truth. For the STARE dataset, the first observer’s manual segmentations are used as ground truth. For the ARIA dataset, the manual segmentation results from the observer DGP are used as ground truth. For the VAMPIRE dataset, the manual segmentations provided are used as ground truth and the images were downsampled to a size of 1,950×1,536 pixels to reduce the computational time.

The segmentation framework was mainly implemented in Matlab version 2013a (Mathworks, Natick, CA) with C++ wrapper code for integration with the C++ implementation of the graph cut segmentation method [48]. All the experiments were performed on a HP Compaq 8200 Elite Small Form Factor PC (3.1GHz Intel Core i3 Processor, 8GB RAM).

### Experiments

Our proposed segmentation framework contains three essential steps: Retinex, local phase-based (LP) enhancement and graph cut-based (GC) segmentation. In order to validate our belief that such a combination is both effective and superior, we performed comparative studies to study the effect of the Retinex, vessel enhancement techniques and segmentation models on the segmentation performance and computational time.The effect of the Retinex enhancement was evaluated by running the two other components of the proposed framework with and without the Retinex enhancement respectively. When evaluating the effect of all the other factors, the Retinex enhancement was always used. For the vessel enhancement approaches, we compared the LP with two other state-of-the-art enhancement approaches: Frangi’s eigenvalue based filter [28] (FR) and the wavelet filter [17] (WL). For the segmentation method, two alternative segmentation methods—level set (LS) and total variation (TV)—each in turn replaced the graph-cut in the segmentation stage. This was done primarily to evaluate whether the graph cut-based method [48] would cause more discretization errors compared to the LS and TV optimization strategies. Thus, there would be in total nine combinations of enhancement and segmentation methods: LP+GC, LP+LS, LP+TV, FR+GC, FR+LS, FR+TV, WL+GC, WL+LS, and WL+TV.

The values of free parameters associated with different filters and segmentation models are tuned for optimal performance using the training data provided by the DRIVE dataset. In this paper, the parameters for each methods are fixed: *λ*_1_ = 3 and *λ*_2_ = 10 for the LS method, *λ*_1_ = 1 and *λ*_2_ = 3 for the TV method, *λ*_1_ = 1 and *λ*_2_ = 4 for the GC method. The iteration process of GC, TV, and LS will be terminated when the iteration step is 30 or the difference between consecutive steps is smaller than 0.001.

### Evaluation of Individual Components

In this section, the effect of Retinex pre-process in the proposed framework is first analyzed. We then analyzed the effect of the filters and segmentation methods, and the datasets on the segmentation performance by grouping all the results together. The computational time of each component and of the whole segmentation process (including both phases of enhancement and segmentation) are also reported.

#### Effect of Retinex

**Fig 5:**
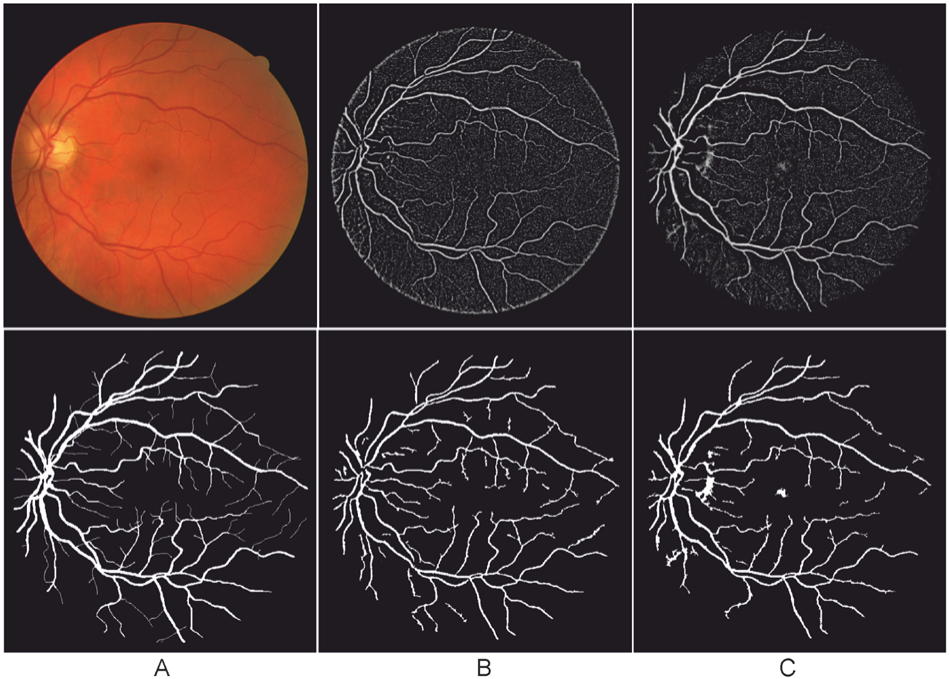
Relative importance of Retinex-based inhomogeneities correction. (A) A randomly chosen image from the DRIVE dataset and expert’s annotation. (B) Vesselness map using local phase filter (top), and the segmentation result (bottom) when the Retinex is applied. (C) Vesselness map using local phase filter (top), and the segmentation result (bottom) when the Retinex is not used.

The optic disk and foveal area in retinal images often cause problem in false detections by most existing vessel segmentation methods [14, 21]. Fig. 5 shows the importance of the Retinex-based filtering on the local phase-based vessel enhancement and graph cut segmentation results. As we mentioned in the previous section, after application of the Retinex the optic disc region has been normalized to a similar level with the background. Therefore, the optic disc will not be enhanced after local phase filtering, and will not be misidentified as a vessel after segmentation, which is a problem when the Retinex is not used. This will lead to higher specificity values when Retinex applies on. Meanwhile, the sensitive scores are very similar irrespective of the presence or absence of the Retinex. Table 1 presents the evaluation results in terms of the proposed framework with and without Retinex pre-processing algorithm applied. It can be seen that Retinex contributes significantly to the final performance results (*Acc* and *AUC*). Overall about 1% of improvement in specificity/accuracy/AUC can be achieved for the color images while little effect on angiographic images. This may be due to the fact that for angiographic images the contrast is already good.

**Table 1:** Segmentation performance of Retinex pre-processing algorithm with and without applied on segmentation framework. *Se*: sensitivity; *Sp*: specificity; *Acc*: accuracy; *AUC*: area under the curve.

**Dataset**	**Retinex**	**Se**	**Sp**	**Acc**	**AUC**
DRIVE	Yes	0.744	0.978	0.953	0.861
	No	0.744	0.963	0.939	0.856
STARE	Yes	0.786	0.975	0.951	0.881
	No	0.787	0.961	0.940	0.876
ARIA	Yes	0.751	0.930	0.940	0.841
	No	0.752	0.921	0.931	0.840
VAMPIRE	Yes	0.721	0.984	0.976	0.853
	No	0.720	0.980	0.974	0.852

#### Effect of Enhancement Methods

**Fig 6:**
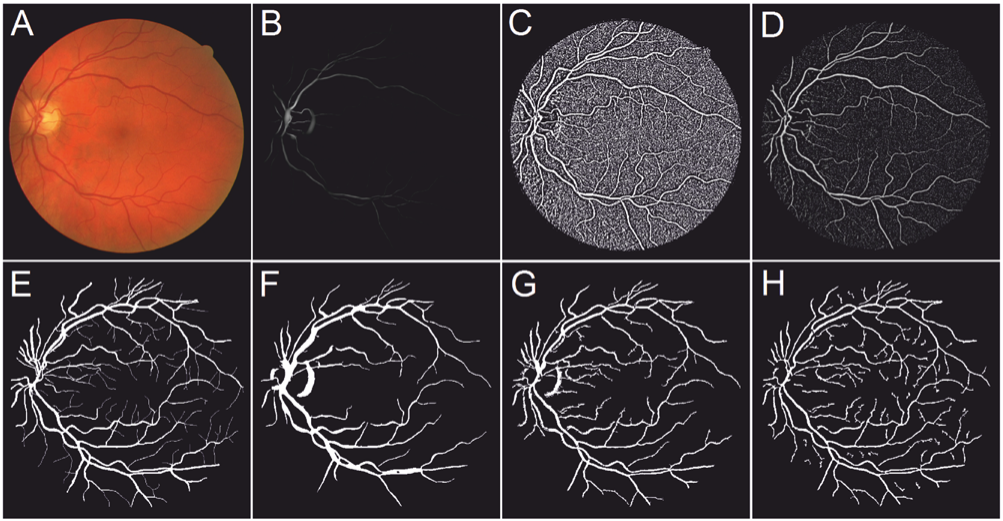
Illustrative enhancement results using different methods and their subsequent graph cut based segmentation results. (A) A randomly chosen image from the DRIVE dataset and expert’s annotation. (B)-(D) Eigenvalue-based (FR), wavelet-based (WL), and proposed local phase-based (LP) enhancements on (A). (E) Expert’s annotation. (F)-(G) Graph cut based segmentation results on (B)-(D).

Fig. 6 shows an example of the segmentation results when a randomly chosen image from the DRIVE dataset was enhanced by the FR, WL, and LP separately, and then segmented using the GC segmentation method on the enhanced images. It can be seen from Fig. 6(B) that the FR tends to only enhance the larger vessels: as does the WL (Fig. 6(C)). The WL also enhanced the non-vessel area (the background), which in turn increase the difficulty of segmentation. As for the proposed LP enhancement results, seen in Fig. 6(D), the edges of the vessels at different scales were enhanced, which made them more stand out more clearly from the background. The segmentation results derived from the FR, WL and LP enhancements are shown in Fig. 6(F)-(H). As expected, the segmentation result based on our LP enhancement showed that more vessels are segmented than is the case in the results based on the other two enhancement methods.

The mean ±standard deviation (STD) of the AUC value is 0.833 ± 0.045, 0.798 ± 0.054 and 0.819 ± 0.036 for the LP, FR and WL, respectively. The difference between these values is statistically significant (ANOVA, *p* < 0.001). The AUC value of the LP is significantly higher than the other two filters (both *p* < 0.001), while the WL outperforms the FR (*p* < 0.001). The mean running time is 17.2 ± 13.7, 44.0 ± 9.0 and 19.5 ± 15.5 seconds for the LP, FR, and WL respectively. Once again, the difference is significant (ANOVA, *p* < 0.001). In particular, the framework using LP is significantly faster than those using the alternative methods (*p* < 0.001 and *p* = 0.01 for the FR and WL respectively), while the WL is significantly faster than the LP implementation (*p* < 0.001).

#### Effect of Segmentation Methods

**Fig 7:**
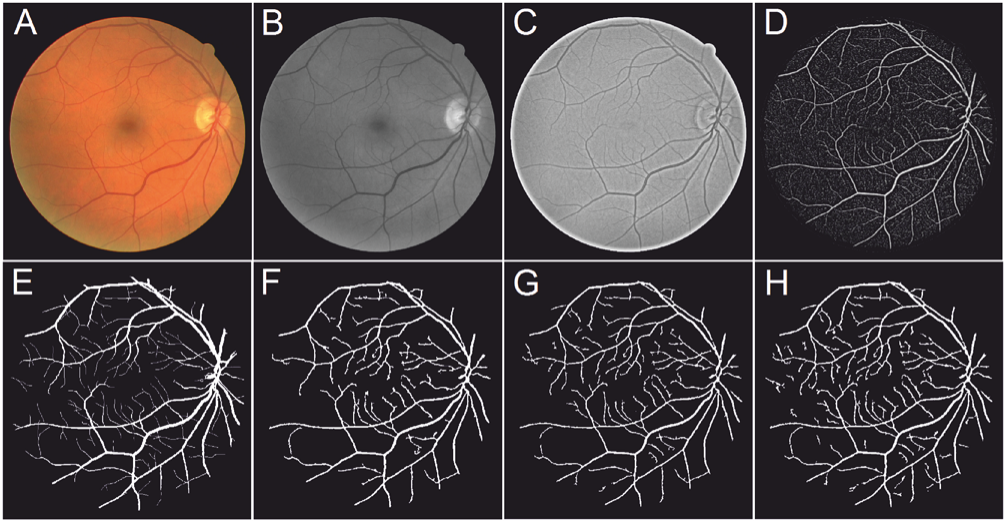
Overview of the main steps of our method and the comparison results obtained with other two segmentation methods. (A) A randomly chosen image from the DRIVE dataset. (B) The green channel of (A): this channel has the highest contrast between regions of vessel and the background. (C) Results after applying Retinex on (B). Retinex successfully enhances the contrast between vessels and background, and the vessels are more easily identifiable. (D) Local phase map of (C): the edges of the vessels are enhanced, and made more visible, to make the vessel stand out further from the background. (E) Expert’s annotation. (F)-(H): Segmentation results with the level set (LS), Total variation (TV), and graph cut (GC) based segmentation methods, respectively.

Fig. 7 (F)-(H) illustrate the segmentation results obtained when a randomly chosen image from the DRIVE dataset is first enhanced by our LP method, then subjected to the LS, TV, and GC segmentation methods, respectively. Fig. 7 (E) is the manual segmentation from the observer. In general, all of these methods are capable of detecting large vessels. However, the GC method (Fig. 7(H)) is more sensitive to the finer vessels, and more vessels have been segmented. Table 2 further confirms this observation: the *Se* has reached the highest value, 0.744, with the combination of LP and GC. In addition, the *Sp* value is 0.978, which is also the highest value in all combinations. Tables 3–5 further indicate that the proposed method outperforms the other combinations on the STARE, ARIA, and VAMPIRE datasets.

**Table 2:** Segmentation performance of different possible combinations of three enhancement methods (LP, WL, FR) and three segmentation methods (GC, TV, LS) on the **DRIVE** dataset. LP, WL and FR denote local phase, wavelet and Frangi’s eigenvalue based enhancement filters respectively. GC, TV and LS denote graph cut, total variation and level set based segmentation methods respectively. *Se*: sensitivity; *Sp*: specificity; *Acc*: accuracy; *AUC*: area under the curve.

**Enhancement**	**Segmentation**	**Se**	**Sp**	**Acc**	**AUC**	**Seg. Time (s)**	**Entire Time (s)**
LP	GC	0.744	0.978	0.953	0.861	0.6	4.6
	TV	0.702	0.949	0.930	0.816	16.1	20.1
	LS	0.679	0.924	0.921	0.802	22.1	26.1
WL	GC	0.744	0.923	0.921	0.833	0.6	5.6
	TV	0.687	0.930	0.912	0.800	16.1	21.1
	LS	0.691	0.934	0.914	0.805	22.1	27.1
FR	GC	0.667	0.921	0.881	0.776	0.6	20.6
	TV	0.722	0.921	0.893	0.807	16.1	36.1
	LS	0.694	0.939	0.927	0.811	22.1	42.1

**Table 3:** Segmentation performance of different possible combinations of three enhancement methods (LP, WL, FR) and three segmentation methods (GC, TV, LS) on the **STARE** dataset. LP, WL and FR denote local phase, wavelet and Frangi’s eigenvalue based enhancement filters respectively. GC, TV and LS denote graph cut, total variation and level set based segmentation methods respectively. *Se*: sensitivity; *Sp*: specificity; *Acc*: accuracy; *AUC*: area under the curve.

**Enhancement**	**Segmentation**	**Se**	**Sp**	**Acc**	**AUC**	**Seg. Time (s)**	**Entire Time (s)**
LP	GC	0.786	0.975	0.951	0.881	0.5	3.5
	TV	0.777	0.960	0.945	0.869	15.2	18.2
	LS	0.775	0.950	0.937	0.863	18.7	21.7
WL	GC	0.640	0.985	0.960	0.813	0.5	4.5
	TV	0.634	0.967	0.948	0.800	15.2	19.2
	LS	0.620	0.960	0.943	0.792	18.7	22.7
FR	GC	0.626	0.976	0.951	0.801	0.5	18.5
	TV	0.423	0.995	0.912	0.709	15.2	33.2
	LS	0.633	0.964	0.938	0.799	18.7	36.7

**Table 4:** Segmentation performance of different possible combinations of three enhancement methods (LP, WL, FR) and three segmentation methods (GC, TV, LS) on the **ARIA** dataset. LP, WL and FR denote local phase, wavelet and Frangi’s eigenvalue based enhancement filters respectively. GC, TV and LS denote graph cut, total variation and level set based segmentation methods respectively. *Se*: sensitivity; *Sp*: specificity; *Acc*: accuracy; *AUC*: area under the curve.

**Enhancement**	**Segmentation**	**Se**	**Sp**	**Acc**	**AUC**	**Seg. Time (s)**	**Entire Time (s)**
LP	GC	0.751	0.930	0.920	0.841	0.6	4.6
	TV	0.712	0.921	0.919	0.817	16.5	20.5
	LS	0.677	0.924	0.921	0.801	22.9	26.9
WL	GC	0.736	0.920	0.912	0.830	0.6	5.6
	TV	0.689	0.922	0.916	0.802	16.5	21.5
	LS	0.695	0.929	0.928	0.811	22.9	27.9
FR	GC	0.717	0.934	0.921	0.776	0.6	19.7
	TV	0.712	0.893	0.921	0.807	16.5	35.6
	LS	0.694	0.927	0.939	0.811	22.9	42.0

**Table 5:** Segmentation performance of different possible combinations of three enhancement methods (LP, WL, FR) and three segmentation methods (GC, TV, LS) on the **VAMPIRE** dataset. LP, WL and FR denote local phase, wavelet and Frangi’s eigenvalue based enhancement filters respectively. GC, TV and LS denote graph cut, total variation and level set based segmentation methods respectively. *Se*: sensitivity; *Sp*: specificity; *Acc*: accuracy; *AUC*: area under the curve.

**Enhancement**	**Segmentation**	**Se**	**Sp**	**Acc**	**AUC**	**Seg. Time (s)**	**Entire Time (s)**
LP	GC	0.721	0.984	0.976	0.853	2.5	12.5
	TV	0.701	0.985	0.976	0.843	30.2	40.2
	LS	0.715	0.984	0.976	0.850	48.3	58.3
WL	GC	0.708	0.975	0.967	0.842	2.5	15.5
	TV	0.687	0.981	0.957	0.813	30.2	43.2
	LS	0.722	0.981	0.960	0.851	48.3	61.3
FR	GC	0.665	0.967	0.957	0.816	2.5	30.5
	TV	0.608	0.980	0.939	0.799	30.2	58.2
	LS	0.705	0.986	0.950	0.846	48.3	76.3

The mean AUC value is 0.818 ± 0.057, 0.817 ± 0.057 and 0.815 ± 0.036 for the GC, TV, and LS implementations respectively. The difference between them is not statistically significant (ANOVA, *p* = 0.67). The mean running time is 13.4 ± 18.8, 28.7 ± 12.5 and 38.6 ± 10.9 seconds for the GC, TV, and LS implementations respectively. This difference is significant (*p* < 0.001). In particular, the GC method is significantly faster than the other two methods (both *p* < 0.001), while the TV method is significantly faster than the LS implementation (*p* < 0.001).

#### Effect of Datasets

The mean values of AUC are 0.816 ± 0.049, 0.817 ± 0.056, 0.813 ± 0.064, and 0.836 ± 0.035 for the ARIA, DRIVE, STARE and VAMPIRE datasets, respectively. There are statistically significant differences in terms of AUC with respect to different datasets (*p* = 0.008). The Tukey post hoc test shows that there is no significant difference between the three color fundus datasets (*p* > 0.8), but that the AUC of the VAMPIRE dataset is significantly higher (*p* = 0.01, 0.03, and 0.005 when compared to the ARIA, DRIVE and STARE). There is also a significant difference in terms of the computational time (ANOVA *p* < 0.001), which is expected as the image size is different across the four datasets. Note, the proposed framework has also been tested on the full sized images of the VAMPIRE dataset: The results are very similar to what we have achieved by downsampling (see details on Table 5), on average the scores in terms of sensitivity, specificity, accuracy and AUC on the full sized images are only 0.002 higher than those of the segmentation results on the downsampled images. However, it took about 50 seconds to segment a full sized image which is almost 4 times longer than the time on downsampled image (13 seconds per image).

These analysis results further confirm that the LP method can provide better segmentation performance with relatively lower computation time: and that the graph cut can provide comparable performance to other segmentation methods, but with significantly shorter time. Therefore, the proposed segmentation framework integrating the Retinex, local phase enhancement with graph cut optimization is the most effective for vessel segmentation.

### Comparison to the Other Methods

By means of the previous experiments we have demonstrated that the proposed framework is both effective and efficient in the task of vessel segmentation. To emphasize the effectiveness of our proposed method, we compared the performance of our method with existing state-of-the-art vessel detection methods on the two most popular public datasets: DRIVE and STARE. The ARIA and VAMPIRE datasets are not used here as they are relatively new, and in consequence there are relatively few results from them in the literature. We chose the most recent six supervised methods [7, 8, 10–13]. Further to this, we selected another seven methods from the unsupervised methods category: [14–17, 19–21]. The results are shown in Table 6. From Table 6, it will clearly be seen that our framework is at least comparable, in performance, even where it does not outperform the other methods. It is worth noting that the methods with stars (*) indicate that a pre-processing step is included in their segmentation frameworks.

**Table 6:** Performance of different segmentation methods, in terms of sensitivity (*Se*), specificity (*Sp*), accuracy (*Acc*) area under the curve (*AUC*), on the **DRIVE** and **STARE** datasets.

Method	**DRIVE**				**STARE**			
	Se	Sp	Acc	AUC	Se	Sp	Acc	AUC
Second observer	0.776	0.972	0.947	0.874	0.895	0.938	0.934	0.917
***Supervised methods***								
Staal et.al [7]	-	-	0.946	-	-	-	0.951	-
Soares et.al [8]*	-	-	0.946	-	-	-	0.948	-
Lupascu et.al [10]	0.720	-	0.959	-	-	-	-	-
You et.al [13]*	0.741	0.975	0.943	0.858	0.726	0.975	0.949	0.851
Marin et.al [11]	0.706	0.980	0.945	0.843	0.694	0.981	0.952	0.838
Wang et.al [12]*	-	-	0.946	-	-	-	0.952	-
***Unsupervised methods***								
Mendonca et.al [15]*	0.734	0.976	0.945	0.855	0.699	0.973	0.944	0.836
Palomera-Perez et.al [20]	0.660	0.961	0.922	0.811	0.779	0.940	0.924	0.860
Matinez-Perez et.al [19]	0.724	0.965	0.934	0.845	0.750	0.956	0.941	0.853
Al-Diri et.al [16]	0.728	0.955	-	0.842	0.752	0.968	-	0.860
Fraz et.al [14]*	0.715	0.976	0.943	0.846	0.731	0.968	0.944	0.850
Nguyen et.al [21]	-	-	0.940	-	-	-	0.932	-
Bankhead et.al [17]	0.703	0.971	0.9371	0.837	0.758	0.950	0.932	0.854
Orlando et.al [23]	0.785	0.967	-	-	-	-	-	-
Azzopardi et.al [22]	0.766	0.970	0.944	0.961	0.772	0.970	0.950	0.956
Proposed method*	**0.744**	**0.978**	**0.953**	**0.861**	**0.786**	**0.975**	**0.951**	**0.881**

The results on the DRIVE dataset show that the sensitivity of the proposed method are in top three in both of the supervised and unsupervised methods, with *Se* = 0.744. The specificity *Sp* = 0.978 and *Acc* = 0.953, which are also the highest value among the unsupervised methods, and only 0.002 and 0.006 behind the supervised method [10, 11]. On the STARE images, our proposed method recorded the best performance in terms of sensitivity, specificity, and accuracy among the unsupervised methods.

## Discussion and Conclusions

In this paper, we have proposed a new framework for the vessel segmentation problem, which exploits the advantages of Retinex-based intensity inhomogeneity correction, local phase-based enhancement, and graph cut-based active contour model. The proposed framework has been applied to four publicly available retinal datasets and the results demonstrated that each components of the framework can provide the level of performance expected, and that the overall framework outperforms most of the existing methods in terms of accuracy and efficiency.

To the best knowledge of the authors this is the first work that a segmentation algorithm has been evaluated on four datasets including both color fundus images and fluorescein angiography images. It is important to note that over the two third of the images used here are from patients with various diseases such as diabetic complications and age-related macular degeneration while the remainders of them are from healthy volunteers. Our results strongly suggested that the proposed framework will be useful in the management of retinal disease. Color fundus images are the only established imaging technique that has been used in the screening of diabetes and also widely used by opticians and in hospitals. Fluorescein angiography is primarily used in the differential diagnosis of retinal disease and treatment planning. Our framework has shown good performance for both imaging modalities. Incorporation of our proposed method of extracting and analysing vasculature promises a wide range of applications. For example, the framework will be applicable to the management of other eye condition such as corneal neovascularization [53].

**Fig 8:**
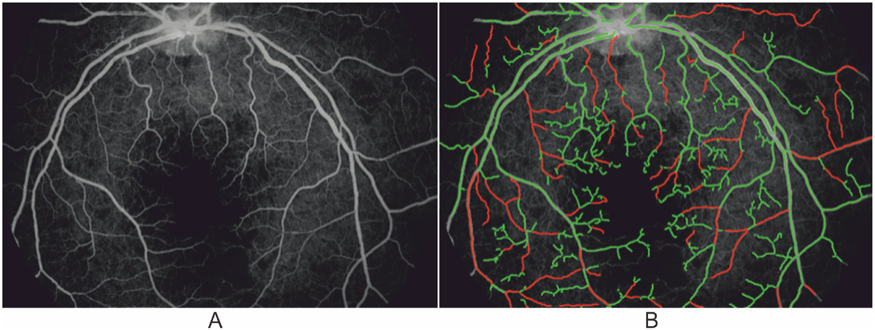
Illustrative vessel abnormality detection result based on the proposed segmentation method. (A) Original fluorescence angiography image. (B) Abnormality detection result. Red color indicates abnormal vessels and green color shows normal vessels.

The detection of vessels essentially is the first but important step for automated vessel analysis tools. After vessel segmentation, it is possible to perform more advanced analysis, such as measurements of diameters and tortuosity of the vessels, classification of veins and arteries, calculation of arteriovenous ratio, and more importantly study the diagnostic and prognostic values of these features on eye disease and a number of systematic diseases (e.g. stroke, hypertension etc). For instance, Fig. 8 shows an example application on the detection of abnormal vessels due to the parasite sequestration in malarial retinopathy based on the proposed framework: here green indicates normal vessels and red abnormal vessels.

Although in this paper we have only evaluated our proposed framework on retinal imagery due to the limited availability of public datasets, the framework is well suited to address segmentation problems in images of other organs acquired using different imaging techniques such as MRI and X-Ray images. There has been increasing use of three-dimensional (3D) images in clinical settings. It is our belief that it would be straightforward to extend our framework to 3D. First, Retinex has been successfully applied to 3D shapes in our previous work [54]. Second, local phase can be defined in 3D space by means of monogenic signal. In particular, here we used optimized lognormal filters to derive the local phase: other quadrature filters, such as the Cauchy filter [45], may equally be used. We expect the possible gain would be relatively small. In addition, filter optimization should be considered in order to achieve good performance in both the frequency and spatial domain. Finally, graph cut-based active contour approach has already demonstrated good performance in 3D image segmentation problems [55].

The program by far is not optimized for speed. As an initiative of reproducible research it is our intention to optimize the code for efficiency and then share the refined source code with the research community in vessel analysis. By doing this we expect more researchers can have access to our programs for their own applications.

In conclusion, in this paper we have proposed an efficient and effective framework for retinal vessel segmentation with good performance. This will become a powerful tool for management of a wide spectrum of vascular-related diseases.
