# Correction: Retinal Vessel Segmentation: An Efficient Graph Cut Approach with Retinex and Local Phase

**DOI:** 10.1371/journal.pone.0127486

**Published:** 2015-04-24

**Authors:** Yitian Zhao, Yonghuai Liu, Xiangqian Wu, Simon P. Harding, Yalin Zheng

The second affiliation for the first author was inadvertently listed as his current address. Yitian Zhao is also affiliated with: School of Mechatronical Engineering, Beijing Institute of Technology, Beijing, China.
